# TGF-β concentrations and activity are down-regulated in the aqueous humor of patients with neovascular age-related macular degeneration

**DOI:** 10.1038/s41598-018-26442-0

**Published:** 2018-05-23

**Authors:** Gian Marco Tosi, Giovanni Neri, Elena Caldi, Fiorella Fusco, Tommaso Bacci, Antonio Tarantello, Elisabetta Nuti, Davide Marigliani, Stefano Baiocchi, Claudio Traversi, Marcella Barbarino, Chiara M. Eandi, Barbara Parolini, Lucia Mundo, Annalisa Santucci, Maurizio Orlandini, Federico Galvagni

**Affiliations:** 1 University of Siena, Ophthalmology Unit of the Department of Medicine, Surgery and Neuroscience, Siena, 53100 Italy; 2 University of Siena, Department of Biotechnology, Chemistry and Pharmacy, Siena, 53100 Italy; 3 University of Siena, Department of Medicine, Surgery and Neuroscience, Siena, 53100 Italy; 4 University of Turin, Department of Surgical Science, Turin, 10124 Italy; 5 Vitreoretinal Unit, Sant’Anna Hospital, Brescia, Italy; 6 University of Siena, Department of Medical Biotechnology, Siena, 53100 Italy

## Abstract

Controversy still exists regarding the role of the TGF-β in neovascular age-related macular degeneration (nAMD), a major cause of severe visual loss in the elderly in developed countries. Here, we measured the concentrations of active TGF-β1, TGF-β2, and TGF-β3 by ELISA in the aqueous humor of 20 patients affected by nAMD, who received 3 consecutive monthly intravitreal injections of anti-VEGF-A antibody. Samples were collected at baseline (before the first injection), month 1 (before the second injection), and month 2 (before the third injection). The same samples were used in a luciferase-based reporter assay to test the TGF-β pathway activation. Active TGF-β1 concentrations in the aqueous humor were below the minimum detectable dose. Active TGF-β2 concentrations were significantly lower at baseline and at month 1, compared to controls. No significant differences in active TGF-β3 concentration were found among the sample groups. Moreover, TGF-β pathway activation was significantly lower at baseline compared to controls. Our data corroborate an anti-angiogenic role for TGF-β2 in nAMD. This should be considered from the perspective of a therapy using TGF-β inhibitors.

## Introduction

Age-related macular degeneration (AMD) is a major cause of visual loss among the elderly in developed countries. Neovascular AMD (nAMD), a subtype of advanced AMD, is responsible for almost 90% of severe visual loss due to AMD^1^. Many growth factors have been implicated in the pathogenesis of nAMD, with a prominent role being played by vascular endothelial growth factor A (VEGF-A)^2^. Transforming growth factor beta (TGF-β) has also been studied extensively. The TGF-β family consists of a group of three isoforms, TGF-β1, TGF-β2, and TGF-β3, with redundant functions. TGF-β is secreted in a latent (not biologically active) form and is unable to bind to receptors. Before TGF-β can exert its biological effects, it needs to be activated by being released from the complex with latency-associated peptide (LAP) and latent transforming growth factor-beta binding protein (LTBP)^3^. Active TGF-β binds specific transmembrane Ser/Thr kinase receptors, which in turn transduce the signal by phosphorylating the receptor-regulated (R-) SMAD2/3 transcription factors.

Controversy still exists regarding the role of the TGF-β family members in choroidal neovascularization (CNV), since they have a pleiotropic effect and may play different roles in the regulation of vascular endothelial and smooth muscle cells, being either anti- or pro-angiogenic in a context-dependent way^4,5^. In the eye, TGF-β has been shown to act as an anti-angiogenic factor^6^ and to protect retinal pigment epithelium (RPE) cells and retinal vasculature^7–10^. However, other findings suggest it has a pro-angiogenic effect in the eye: TGF-β not only induces VEGF-A expression by RPE^11^, but its direct and indirect inhibition has also been linked to the inhibition of both the epithelial-mesenchymal transition and senescence of RPE cells *in vitro*^12–15^, as well as to the blockage of CNV^16–18^, including the subretinal fibrosis stage^19,20^, in animal models. In humans an increased expression of TGF-β has been documented in surgically removed CNV tissue^21^, while an increased concentration of total TGF-β1 has been found in the vitreous^18^ and aqueous humor^22^ of nAMD patients. Thus, in accordance with the possible role of TGF-β in favoring CNV, its treatment with TGF-β blocking agents has been advocated^16–20^.

However, knowledge in this field is still limited, since the vast majority of the above-mentioned studies (regarding both anti- and pro-angiogenic aspects) have been conducted *in vitro* or in animals, and rarely consider all the TGF-β isoforms simultaneously. In fact, in addition to TGF-β1, TGF-β2, which is presumed to be the predominant isoform in the eye^15,19,23^, and TGF-β3 might deserve attention in the context of nAMD, where they have not been measured yet. Moreover, measurement of the total TGF-β1 protein concentration in the biological fluid of human eyes, as previously conducted by our group^22^ and by Bay *et al*.^18^, might only partially reflect the effective concentration of the active form.

In the present study we evaluated the protein concentration of active TGF-β1, TGF-β2, and TGF-β3 in the aqueous humor of patients affected by naïve nAMD, at baseline and after intravitreal anti-VEGF-A injection. Moreover, we set up a luciferase-based reporter assay to test the TGF-β pathway activation by aqueous humor samples.

## Results

### Assessment of active TGF-β1, β2 and β3 levels in aqueous humor

The study measured the levels of active TGF-β1, β2 and β3 in the aqueous humor of 20 patients with nAMD, who received 3 consecutive monthly intravitreal injections of anti-VEGF-A antibody (0.5 mg ranibizumab). Samples were collected at baseline (before the first injection), month 1 (before the second injection), and month 2 (before the third injection). 20 age-matched cataract patients served as controls. The demographic data and patient characteristics are stated in Table 1. Concentrations of active TGF-βs were measured by ELISA. Active TGF-β2 and β3 were detected and quantified both in controls and in patients, while the active TGF-β1 concentration was below the minimum detectable dose (5 pg/ml) for the majority of the samples analyzed (data not shown).

**Table 1:** Comparison of nAMD patients and control group, with patients’ clinical characteristics.

	nAMD Group, n = 20	Control Group, n = 20	*P* value
Age (years)	77.5 ± 10.25	77.65 ± 8.68	0.578^#^
Sex (male/female)	1:0.67	0.82:1	0.342^§^
Eyes (n)	20	20	N/A
Type of choroidal neovascularization (n)	16 type 1 4 type 2	N/A	N/A
Central Retinal Thickness day 0 (μm)	306.45 ± 57.78	N/A	N/A
Central Retinal Thickness after 3 treatments (μm)	239.45 ± 33.85	N/A	N/A
Neovascular lesion size (μm)	1971.25 ± 1021.02	N/A	N/A

Age, retinal thickness and lesion size are expressed as mean ± standard deviation. N/A: not applicable. ^#^Mann-Whitney U test. ^§^Chi-squared test.

**Figure 1:**
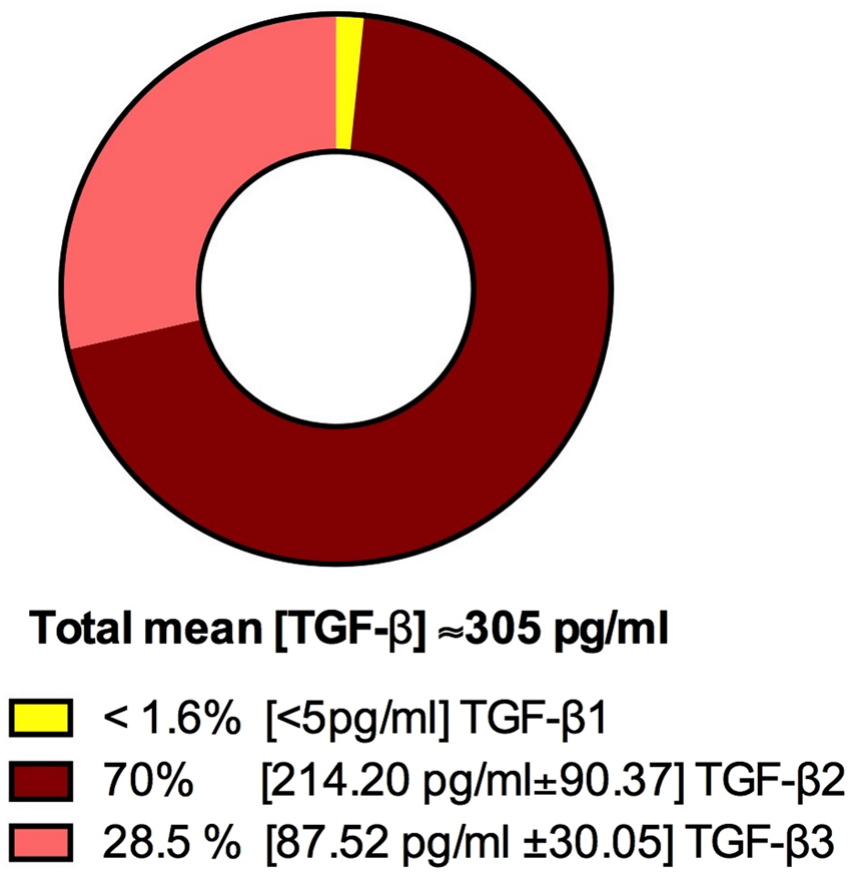
Pie chart of TGF-β1, TGF-β2 and TGF-β3 concentrations in the aqueous humor of a sample group of 20 healthy subjects. The concentration of active TGF-β1, β2 and β3 was measured by ELISA. The mean of the total concentration of all TGF-βs, the percentage of the total, and the mean ± SD of each isoform are shown. Each sample was measured twice for TGF-β2 and TGF-β3 and once for TGF-β1.

In controls, the aqueous humor concentration of active TGF-β revealed the preponderance of TGF-β2 over TGF-β3 and, to a greater extent, over TGF-β1. In particular, from total active TGF-β, 70% was TGF-β2, 28.5% was TGF-β3 and less than 1.6% was TGF-β1, thus suggesting an important role of TGF-β2 and TGF-β3 in the homeostasis of the eye (Fig. 1).

**Figure 2:**
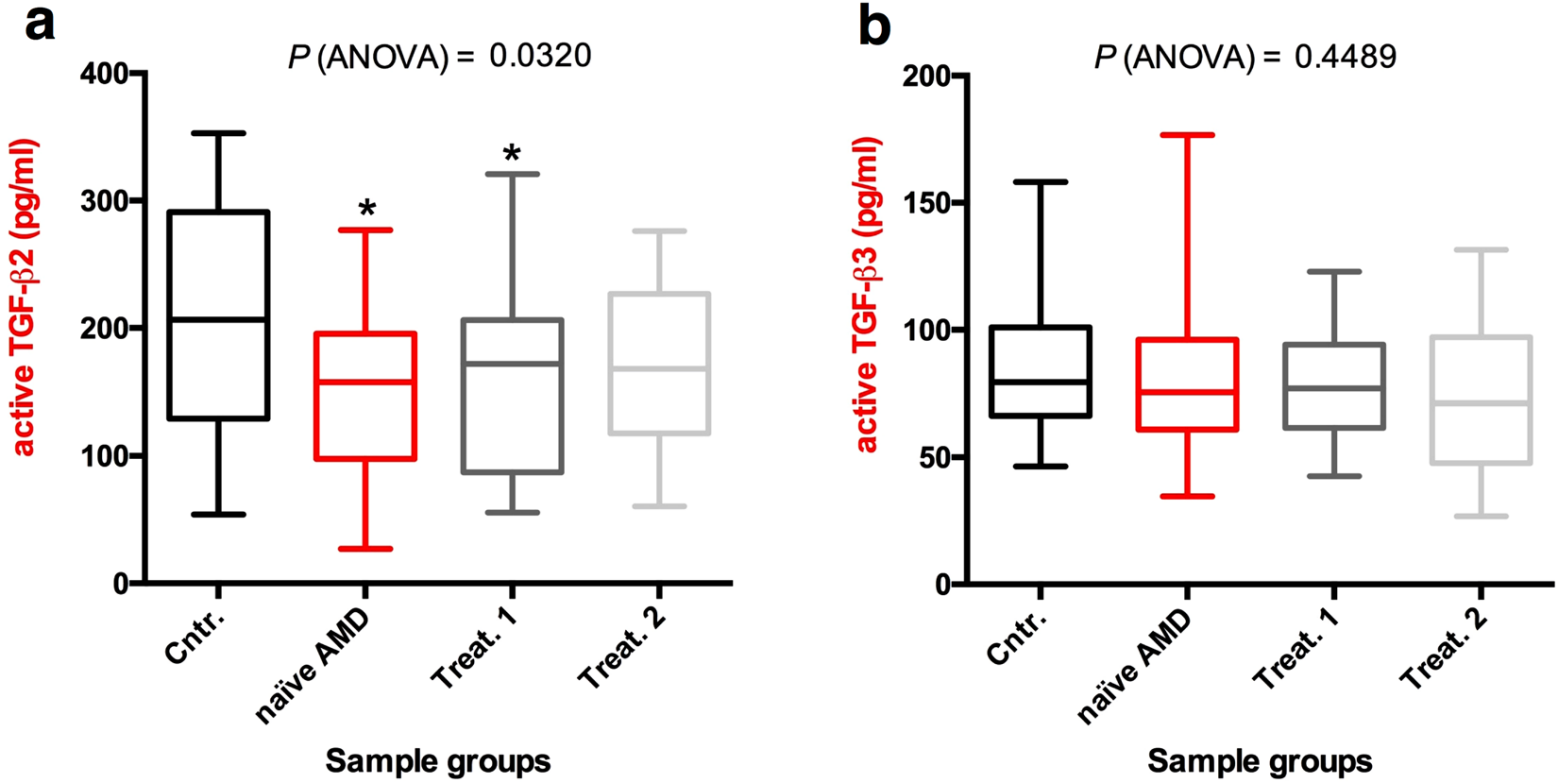
ELISA analysis of the aqueous levels of TGF-β2 and TGF-β3. The TGF-β2 (**a**) and TGF-β3 (**b**) protein concentrations in the aqueous humor of 20 patients and 20 controls (described in Table 1) were determined by ELISA. Each sample was measured twice. Data are presented as box and whisker plots displaying the median, lower, and upper quartiles (boxes) and minimum-maximum (whiskers). Asterisks indicate significant differences (*P < 0.05) between naïve or treated (once or twice, Treat.1 and Treat.2, respectively) patient groups and the control group (Cntr.).

**Figure 3:**
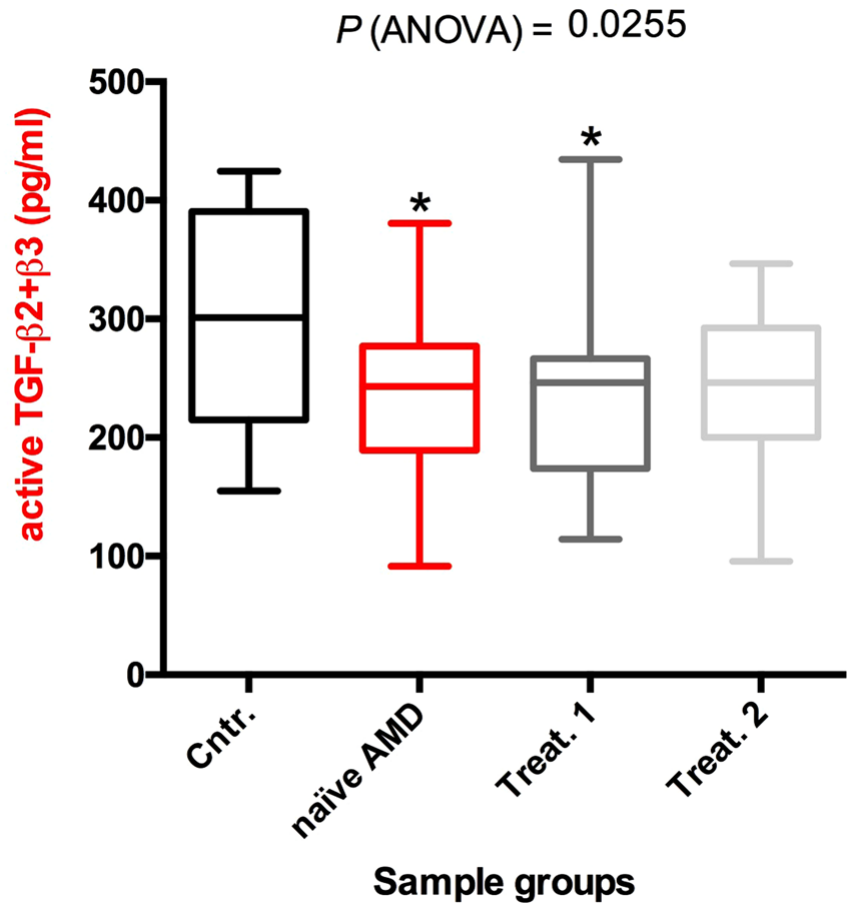
Box and whisker plot showing the sum of TGF-β2 and TGF-β3 concentrations for every single sample (derived from dataset of Fig. 2). Data are presented as box and whisker plots displaying the median, lower, and upper quartiles (boxes) and minimum-maximum (whiskers). Asterisks indicate significant differences (*P < 0.05) between naïve or treated (once or twice, Treat.1 and Treat.2, respectively) patient groups and the control group (Cntr.).

The comparison of active TGF-β2 and TGF-β3 concentrations between patients and controls, revealed that baseline (naïve AMD) and month 1 (Treat.1) concentrations of active TGF-β2 were lower than in controls (controls mean ± SD = 214.2 ± 90.37 pg/mL; baseline AMD mean = 151.4 ± 63.6 pg/mL; month 1 mean = 157.3 ± 71.0 pg/mL), while the month 2 (Treat.2) value was lower, albeit not significantly, than the control value (month 2 mean = 170.7 ± 60.2 pg/mL) (Fig. 2a). The baseline, month 1and month 2 active TGF-β3 concentrations did not differ from controls (controls mean ± SD = 87.5 ± 30.05 pg/mL; baseline mean ± SD = 85.5 ± 39.65 pg/mL; month 1 mean ± SD = 78.6 ± 20.23 pg/mL; month 2 mean ± SD = 73.4 ± 29.61 pg/mL] (Fig. 2b). Additionally, summing the concentrations of active TGF-β2 and TGF-β3 for every single sample, we found that the baseline and month 1 total active TGF-β2/β3 concentration was still significantly lower compared to the control group (controls mean ± SD = 301.8 ± 92.9 pg/mL; baseline AMD mean = 236.9 ± 76.9 pg/mL; month 1 mean ± SD = 235.8 ± 75.1 pg/mL) while month 2 values were slightly, but not significantly, lower than in the control group (month 2 mean ± SD = 244.1 ± 64.73 pg/mL) (Fig. 3).

### Analysis of TGF-β pathway activation

**Figure 4:**
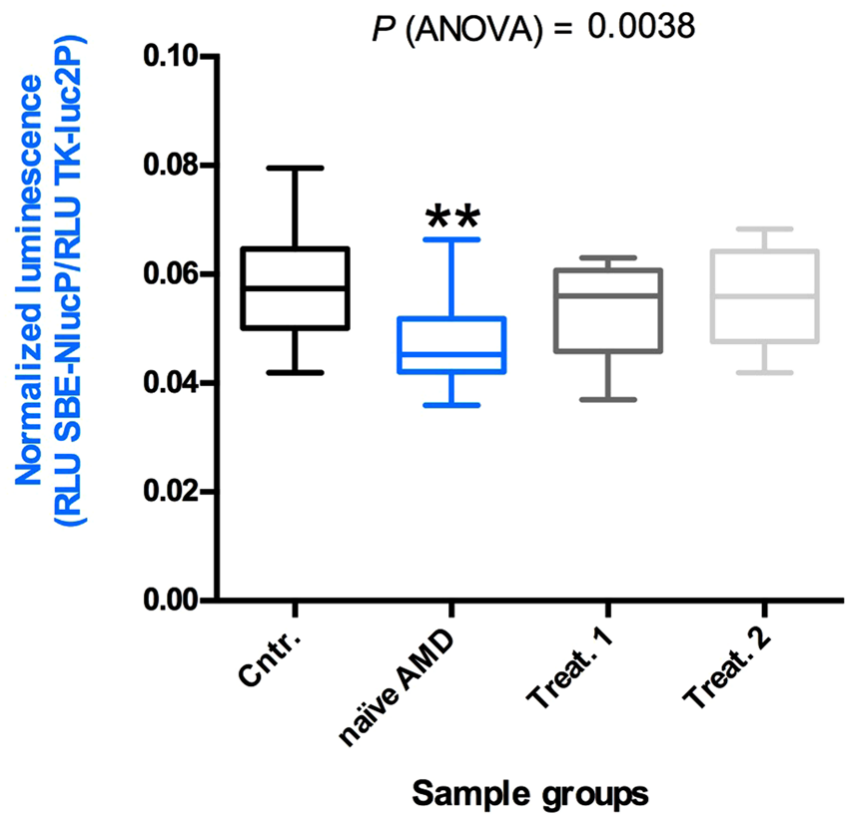
Quantitative analysis of TGF-β pathway activation by aqueous humor of patients and controls. Box and whisker plot showing that the aqueous humor of naïve nAMD patients induces less TGF-β pathway activation in Lenti-X 293T cells. Cells were co-transfected with the plasmids pNL[NlucP/SBE], expressing NanoLuc luciferase under the control of three SMAD3 binding elements, and pGL4.54[luc2/TK], expressing the Firefly luciferase luc2 (used as transfection normalizer) under the control of the constitutive HSV-TK promoter. Transfected cells were treated with 10 μl of aqueous humor of the 20 nAMD patients, naïve or treated (once or twice, Treat.1 and Treat.2, respectively), or of the 20 control samples (Cntr.) described in Table 1. Data are presented as the ratio between NanoLuc and luc2 luciferase activity, expressed as Relative Light Units (RLU) (number of replicates for each sample = 3). Asterisks indicate significant differences (**P < 0.01) between naïve or treated (once or twice, Treat.1 and Treat.2, respectively) patient groups and the control group (Cntr.).

**Figure 5:**
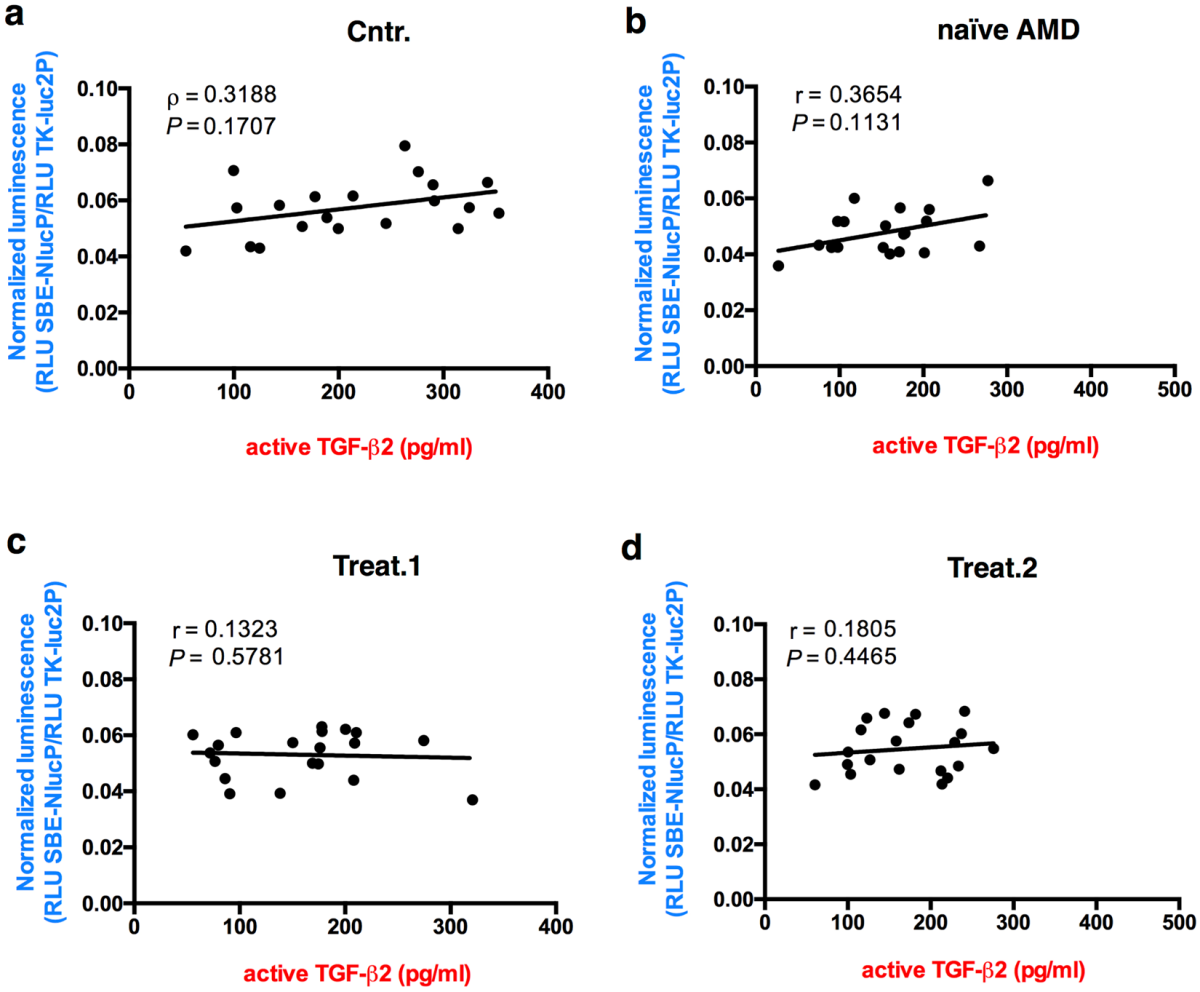
Correlation analysis of TGF-β2 concentrations in the aqueous humor and luciferase activity. Data are presented as scatter plots. No correlation was found between the concentrations of TGF-β2 and SMAD2/3 activation, expressed as luciferase activity. The significance of correlations was tested using Spearman rank-order correlation coefficient (r). *P* (two-tailed) values are indicated.

**Figure 6:**
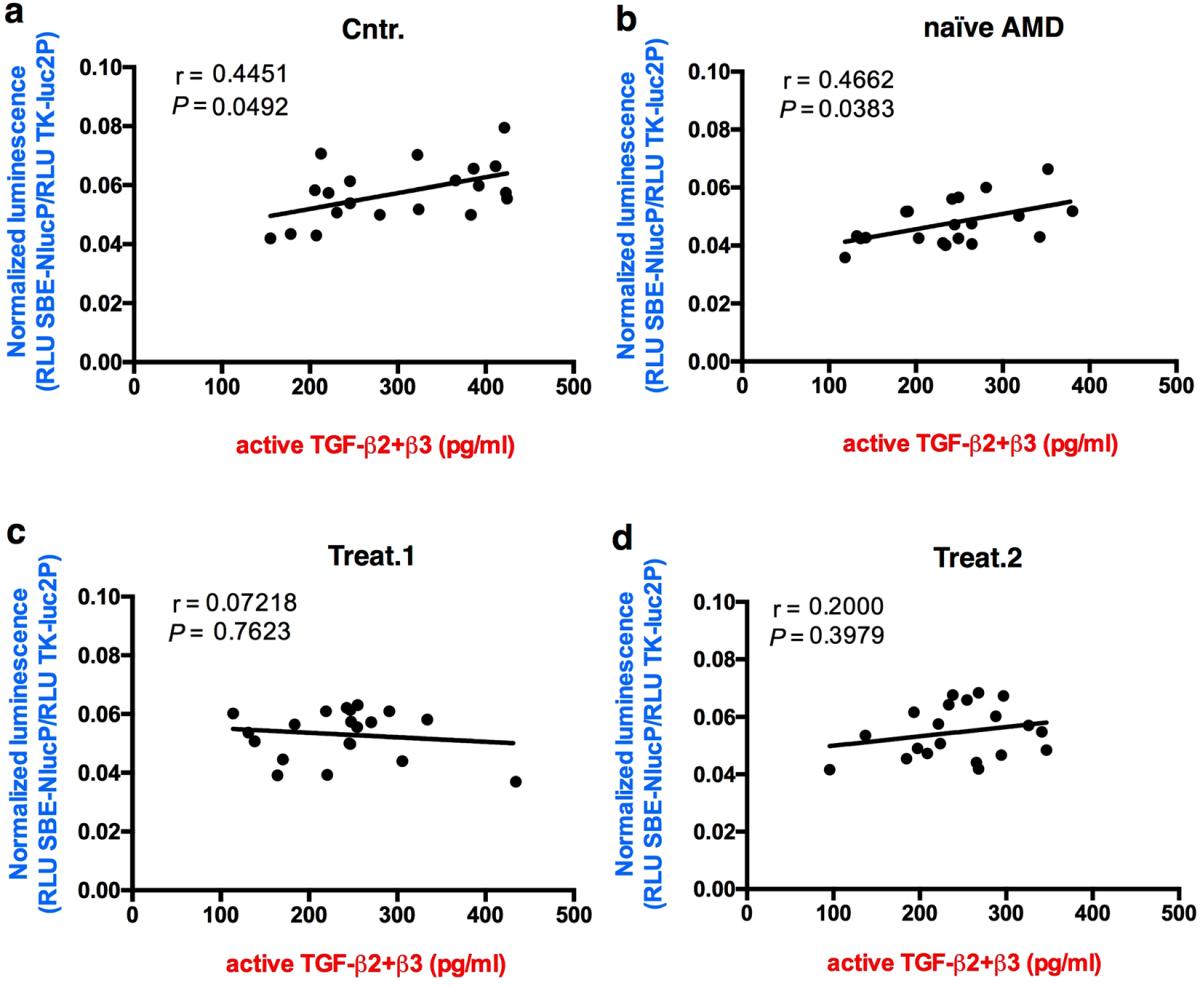
Correlation analysis of total TGF-β2/β3 concentrations in the aqueous humor and luciferase activity. Data are presented as scatter plots. In controls (**a**) and naïve AMD patients (**b**), a correlation was found between the concentrations of total TGF-β2/β3 and SMAD2/3 activation, expressed as luciferase activity. The significance of correlations was tested using Spearman rank-order correlation coefficient (r). *P* (two-tailed) values are indicated. No correlation was found in the treated sample groups (**c**,**d**).

**Figure 7:**
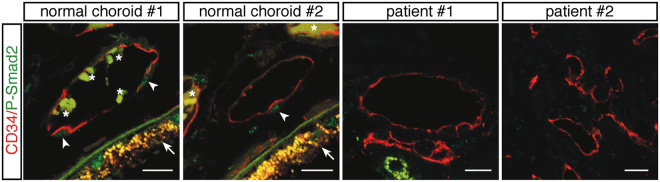
Phospho-SMAD2 (Ser465/467) (P-SMAD2) is detected in the nuclei of ECs of normal choroidal blood vessels but not in ECs of the CNVMs of AMD patients. Fixed paraffin-embedded CNVM sections from 2 AMD patients and human eye sections containing normal choroidal blood vessels were analyzed by immunofluorescence using phospho-SMAD2 (green) and CD34 (red) antibodies. Arrowheads indicate P-SMAD2 positive nuclei. Arrows and asterisks indicate the retinal pigment epithelium and red blood cells, respectively, which exhibits autofluorescence. Scale bars, 14 μm.

The TGF-β superfamily includes, besides TGF-βs, a large group of proteins (BMPs, GFDs, activins, etc) and its signaling is regulated by soluble binding proteins such as Alpha-2-Macroglobulin, Follistatin and Decorin^24^. Therefore, to measure the effective TGF-β pathway activation, we set up a Luciferase-based reporter assay using Lenti-X 293T cells transfected with a plasmid carrying the NanoLuc^®^ gene under the transcriptional control of three copies of the SMAD binding element (SBE) specific for SMAD3^25^. A plasmid for Firefly (luc2) Luciferase expression under the control of the constitutive HSV-TK promoter was also co-transfected as transfection normalizer. The Lenti-X 293T cell line was chosen because it responds to TGF-β stimulation, grows densely and is easy to efficiently transfect, permitting the setup of a sensitive method for measuring TGF-β activity. The transfected cells were treated with 10 μl of aqueous humor of the 20 nAMD patients, naïve or treated, or of the 20 control samples. After 3 hours Firefly and NanoLuc luciferase activities were measured for each sample. We measured a significant reduction in luciferase activity in the cells treated with naïve nAMD samples compared to control samples. In the aqueous humor of the ranibizumab-treated patients, the control level was restored after the first injection (Fig. 4). Although we did not observe a significant correlation between active TGF-β2 concentration and luciferase activity (Fig. 5), we found a significant correlation between the luciferase activity and the sum of the concentrations of active TGF-β2 and TGF-β3 for every single sample in both control and naïve nAMD specimens (Fig. 6a and b), but not in ranibizumab treated patients, both after the first and the second injection (Fig. 6c and d). To evaluate whether lower TGF-β levels in aqueous humor samples of nAMD patients correspond to a down-regulation of the TGF-β pathway activation in choroidal neovascular membranes, we analyzed by immunofluorescence staining the presence of phospho-SMAD2 in endothelial cells (ECs) of normal choroid and choroidal neovascular membranes (CNVMs) obtained from two additional naïve nAMD patients during submacular surgery. As shown in Fig. 7, we observed the phosphorylation of serines 465 and 467 of SMAD2 in ECs of normal choroidal vessels but not of CNVMs from naïve nAMD patients.

## Discussion

Controversy still exists regarding the role of the TGF-β family and its pathway in nAMD. Studies on humans are needed since most of the conflicting evidence comes from *in vitro* or animal studies, which have mainly addressed TGF-β1 and only rarely TGF-β2 and TGF-β3^2,6–11^. Moreover, since the TGF-β isoforms are secreted in their latent form, measurements of their total concentration, as performed in previous human studies, may not necessarily be equivalent to measurements of the active isoforms.

TGF-β1 is the most extensively studied isoform both in *in vitro* and *in vivo*, since it has been found to target the vascular endothelium in normal physiological circumstances to a greater extent than TGF-β2, and to be the isoform most expressed in ECs during embryogenesis^3,26^. Additionally, TGF-β1 has been found to be overexpressed in surgically excised CNV membranes^21^. However, TGF-β2 represents the preponderant isoform in the eye^15,19,23^ and TGF-β2 knockout mice present severe ocular anomalies in the retina, cornea and the lens^23^, thus postulating the isoform’s active involvement in the maintenance of physiological ocular status. Extensive studies in the skin have revealed a role for TGF-β2 and β3 in non-scarring wound healing. Furthermore, it was shown that changes in the ratio between the three isoforms can determine the final anatomical status after inflammation^27,28^. Moreover, although TGF-β1, β2 and β3 derive from different genes, functional redundancy may occur^3^.

The present study analyzes the aqueous humor concentrations of active TGF-β1, β2 and β3 at baseline and after 1 and 2 intravitreal injections of ranibizumab in previously untreated patients affected by nAMD. The same measurements were performed on controls. In controls, the aqueous humor concentration revealed the preponderance of TGF-β2 over TGF-β3 and, to a greater extent, over TGF-β1, thus suggesting a main role for TGF-β2 in the homeostasis of the eye under physiological conditions. This was confirmed under pathological conditions by the lower TGF-β2 concentration measured in nAMD patients at baseline and after injections in comparison to controls (although the difference was not significant at month 2), while the TGF-β3 concentration was unchanged. Considering the total concentration of both these two isoforms in each of the patients, we obtained a trend similar to that of TGF-β2 concentration alone, with the baseline concentration still significantly lower compared to the control group. This shows that the preponderant TGF-β2 has an important effect on the final total concentration and could suggest a protective effect of TGF-β2 on nAMD. In accordance with our results, Yafai and colleagues showed a Müller cell-guided inhibition of retinal ECs proliferation via TGF-β2^29^. On the contrary, in animal models, Zhang *et al*. showed that the inhibition of TGF-β^20^ and the antagonism of COX-2^19^, with the consequent down-regulation of TGF-β2, attenuated subretinal fibrosis after CNV induction. However, the subretinal fibrosis stage in their CNV animal model can hardly be compared to our findings regarding the first stage of CNV in humans. In accordance with Zhang *et al*.^19,20^. Recalde and colleagues showed a reduction of diode-laser-induced CNV in rats following use of two peptides anti-TGF-β, including TGF-β2^17^. However, the laser-induced CNV animal model is characterized by an acute injury and inflammation, representing only the neovascular component of the disease, and consequently is unable to recapitulate the complex sequence of events leading to the development of CNV in patients with nAMD, such as long-term senescent degeneration and chronic inflammation.

We measured TGF-β in the active form but, since the TGF-β pathway is context-specific and potentially influenced by many factors, we also wanted to verify whether there was a correlation between the concentrations of the active isoforms and the activity of the pathway. The TGF-β pathway is complex. The three isoforms, together with the other superfamily members, such as the Bone Morphogenetic Proteins (BMP), can interact with two types of receptors (ALK5 and ALK1) and two types of coreceptors (endoglin and betaglycan), leading to the phosphorylation of SMAD2 and 3 - the anti-angiogenic TGF-β/ALK5 pathway - or of SMAD1, 5 and 8 - the proangiogenic TGF-β/ALK1 pathway^3,4,26^. Endoglin seems to play a role in the activation of the ALK1 pro-angiogenic pathway in the presence not only of BMP but also of TGF-β1 and β3^3,4,26^. However, TGF-β1 and 3 can also activate the ALK5/SMAD2 and 3 pathway (the canonical most-studied pathway), leading to an anti-angiogenic effect^3,4,26^. In contrast, TGF-β2 associates with coreceptor betaglycan activating anti-angiogenic ALK5/SMAD2/3 signaling^30–32^. Here, we observed a decrease in SMAD2/3 pathway activation by the aqueous humor of nAMD patients, suggesting a possible down-regulation of the anti-angiogenic TGF-β pathway in nAMD, as also supported by the immunofluorescence data in choroidal ECs. Since in the same patients, we observed a lower TGF-β2 concentration compared to controls, we would have expected a correlation (in each patient) between TGF-β2 concentrations and the decreased SMAD2/3 pathway activation. Surprisingly, SMAD2/3 pathway activation did not correlate with TGF-β2 concentration, but did correlate with the sum of the concentrations of active TGF-β2 and TGF-β3. This suggests that the effect on the TGF-β pathway activation is mediated by the combined action of TGF-β2 and TGF-β3, although only TGF-β2 was down-modulated in nAMD group and consequently could be involved in pathophysiology of nAMD. This correlation was observed in both controls and patients at baseline, but was not apparent after treatment. This may be explained by the cross-talk between the VEGF-A and TGF-β signaling pathways, although not yet completely elucidated^4,33^. Ranibizumab might also change the aqueous molecular constitution via VEGF-A antagonism, thus rendering such a TGF-β pathway less directly dependent on TGF-β levels.

Inhibition of the TGF-β pathway has been advocated as an additional treatment option in the battle against nAMD. In light of our results, which suggest a protective role of TGF-β2 in nAMD, a general inhibition of TGF-β should be viewed with caution, and more specific target should be considered^2^. However, further independent studies are needed to confirm this issue using a larger number of samples and to better understand the functional consequences of different levels of TGF-β pathway activation in nAMD and the distribution of the TGF-β isoforms in the different eye compartments and tissues.

## Methods

### Subjects

This observational case-control study comprised 20 patients affected by active nCNV secondary to AMD. All eyes were examined and treated between June 2016 and December 2016 at the Ophthalmology Unit of the Department of Medicine, Surgery and Neuroscience, Siena University Hospital, Siena, Italy, following approval from the institutional review board. The study complied with the Declaration of Helsinki and was registered in the ISRCTN registry (reference number: ISRCTN60434145; date of registration: 12/01/2018). Patients were treated after being informed of the nature, purpose, implications and risks of the treatment and after having signed a consent form.

The study enrolled patients who presented with CNV secondary to AMD. The diagnosis was confirmed by fluorescein angiography (FA), indocyanine green angiography (ICGA) and spectral domain optical coherence tomography (OCT).

Patient demographics, study eye characteristics and treatment details were recorded, including: best corrected visual acuity (BCVA) at baseline and during the course of follow up, measured using ETDRS charts at a distance of 4 m; CNV subtype; lesion size, determined by the greatest linear dimension measured by fluorescein angiography examination prior to treatment; and central macular thickness (CMT) at baseline and each follow up visit.

None of the patients had received any previous treatment for nAMD, nor had they undergone any previous ophthalmic surgery, except cataract removal. Cataract surgery had to have been performed at least 9 months prior to inclusion. Controls were age-matched patients undergoing cataract surgery. The exclusion criteria for controls were any ocular disease other than cataracts and any previous ophthalmic surgery. Diabetes mellitus, use of immunosuppressive drugs and a malignant tumor in any location constituted exclusion criteria for both patients and controls.

### Aqueous humor sample collection

All patients with nAMD received 3 consecutive monthly intravitreal injections of anti-VEGF-A therapy (ranibizumab 0.5 mg). Aqueous samples were collected prior to injections at baseline (day of the first injection), month 1 (day of the second injection), and month 2 (day of the third injection).

Anterior chamber taps were performed in the operating room prior to each intravitreal injection (patients) and before cataract surgery (controls). A 30-gauge needle was inserted into the anterior chamber and 0.16–0.2 mL of aqueous was collected, centrifuged at 1500 g for 20 minutes to remove cells and debris, aliquoted and frozen at −80 °C until analysis.

### Assessment of active TGF-β1, β2 and β3 levels in aqueous humor

The concentration of active TGF-β1, β2 and β3 in the aqueous humor was measured by enzyme-linked immunosorbent assay (ELISA) using ELISA kits for human TGF-β1, TGF-β2 (Quantikine ELISA kits #DB100B and #DB250, respectively; R&D Systems, Minneapolis, USA) and TGF-β3 (#SEB949Hu; Cloud-Clone Corp., Houston, TX). Each assay was performed according to the manufacturer’s instructions without the acidification step for activation of latent TGF-β, allowing the detection of the active forms. For each measurement of TGF-β concentration, 25 μl of sample were diluted to 100 μl using sample diluent buffer immediately prior to the assay. Each sample was measured twice for TGF-β2 and TGF-β3. Each sample was measured once for TGF-β1, after a preliminary analysis on extra-groups specimens showing that the active TGF-β1 concentration was below the minimum detectable dose (5 pg/ml).

### Analysis of TGF-β pathway activation

The luciferase-based reporter system used to analyze activation of the TGF-β pathway consisted of Lenti-X 293T cells (#632180; Takara Bio, CA) transiently transfected with the plasmid pNL[NlucP/SBE] (Promega Corp., Madison, WI), carrying three copies of the SMAD binding element (SBE) (AGTATGTCTAGACTGA) which represent specific targets of for phospho-SMAD3^25^ and drive the expression of a reporter gene encoding NanoLuc^®^ Luciferase. The plasmid pGL4.54[luc2/TK] (Promega Corp., Madison, WI), carrying the reporter luc2 gene coding firefly luciferase under the control of the constitutive HSV-TK promoter, was co-transfected with pNL[NlucP/SBE] at a molar ratio of 5:1 (pGL4.54[luc2/TK]:pNL[NlucP/SBE]) as a normalizer for transfection efficiency. Cells were grown as previously described^34^ and transfected in suspension using Attractene Transfection reagent (#301005; Qiagen, Hilden, Germany) following the manufacturer’s instructions, then plated (2 × 10^4^ cells/well) on 96-well plates. After 24 hours cells were treated with 10 μl of aqueous humor for 3 hours. Cells were then lysed in 100 μl Passive Lysis Buffer and subsequently 20 μl of the lysate was assayed using the Nano-Glo® Dual-Luciferase® Reporter Assay System (#N1610; Promega Corp., Madison, WI). Luciferase activity was read using TD20/20 luminometer (Turner Diagnostics, Sunnyvale, CA) and expressed as relative luminescence units (RLU). Normalization of transfection efficiency was obtained dividing RLU from NanoLuc^®^ readings by RLU from luc2 readings. Three replicates were performed for each sample.

### Immunofluorescence

CNVMs were collected from naïve nAMD patients during submacular surgery as previously described^35^. Normal choroid sections were obtained from 2 eyes enucleated for retinoblastoma^35^. Specimens were incubated with rabbit polyclonal anti-phospho-Smad2 (Ser465/467) (#AB3849; Chemicon International/Millipore, Billerica, MA) and mouse monoclonal anti-CD34 (#QBEnd/10; Ventana Medical Systems, Inc., Strasbourg, France) as EC marker. Alexa Fluor-488 or 568 (Thermo Fisher Scientific, Waltham, MA) secondary antibodies were used. Fluorescent images were captured using a Leica TCS SP2 laser-scanning confocal microscope.

### Statistical analysis

The data analysis was performed using Prism 6 statistical software (GraphPad Software Inc., San Diego, CA). Data are presented as box and whisker plots displaying the median, lower and upper quartiles and the minimum-maximum. Data normality was tested using D’Agostino and Pearson omnibus normality test. Evaluation of the data was conducted using one-way ANOVA, followed by Bonferroni multiple comparison post-hoc test. The significance of correlations between protein concentrations in the aqueous humor and normalized luminescence was tested using Spearman rank-order correlation coefficient (r). *P* (two-tailed) values are indicated.

### Data Availability

The datasets generated during the current study are available from the corresponding author on reasonable request.
