# Publisher Correction: TGF-β concentrations and activity are down-regulated in the aqueous humor of patients with neovascular age-related macular degeneration

**DOI:** 10.1038/s41598-018-28203-5

**Published:** 2018-06-29

**Authors:** Gian Marco Tosi, Giovanni Neri, Elena Caldi, Fiorella Fusco, Tommaso Bacci, Antonio Tarantello, Elisabetta Nuti, Davide Marigliani, Stefano Baiocchi, Claudio Traversi, Marcella Barbarino, Chiara M. Eandi, Barbara Parolini, Lucia Mundo, Annalisa Santucci, Maurizio Orlandini, Federico Galvagni

**Affiliations:** 1 University of Siena, Ophthalmology Unit of the Department of Medicine, Surgery and Neuroscience, Siena, 53100 Italy; 2 University of Siena, Department of Biotechnology, Chemistry and Pharmacy, Siena, 53100 Italy; 3 University of Siena, Department of Medicine, Surgery and Neuroscience, Siena, 53100 Italy; 4 University of Turin, Department of Surgical Science, Turin, 10124 Italy; 5 Vitreoretinal Unit, Sant’Anna Hospital, Brescia, Italy; 6 University of Siena, Department of Medical Biotechnology, Siena, 53100 Italy

Correction to: *Scientific Reports* 10.1038/s41598-018-26442-0, published online 23 May 2018

The Article contains errors in the Reference list. The order of References 28 – 35 is incorrect. The correct order of References 28-35 appears below.

28) Saed, G. M., Collins, K. L. & Diamond, M. P. Transforming growth factors beta1, beta2 and beta3 and their receptors are differentially expressed in human peritoneal fibroblasts in response to hypoxia. *Am. J. Reprod. Immunol*. **48**(6), 387-93 (2002).

29) Yafai, Y. *et al*. Müller glial cells inhibit proliferation of retinal endothelial cells via TGF-β2 and Smad signaling. *Glia* **62**(9), 1476-85 (2014).

30) López-Casillas, F., Payne, H. M., Andres, J. L. & Massagué, J. Betaglycan can act as a dual modulator of TGF-beta access to signaling receptors: mapping of ligand binding and GAG attachment sites. *J. Cell. Biol*. **124**(4), 557-68 (1994).

31) Sankar, S., Mahooti-Brooks, N., Centrella, M., McCarthy, T. L. & Madri, J. A. Expression of transforming growth factor type III receptor in vascular endothelial cells increases their responsiveness to transforming growth factor beta 2. *J. Biol. Chem*. **270**(22), 13567-72 (1995).

32) Yamashita, H. *et al*. Endoglin forms a heteromeric complex with the signaling receptors for transforming growth factor-beta. *J. Biol. Chem*. **269**(3), 1995-2001 (1994).

33) Yamauchi, K. *et al*. Vascular endothelial cell growth factor attenuates actions of transforming growth factor-beta in human endothelial cells. *J. Biol. Chem*. **279**(53), 55104-8 (2004).

34) Galvagni, F. *et al*. CD93 and dystroglycan cooperation in human endothelial cell adhesion and migration adhesion and migration. *Oncotarget* **7**(9), 10090-103 (2016).

35) Tosi, G. M. *et al*. CD93 as a Potential Target in Neovascular Age-Related Macular Degeneration. *J. Cell. Physiol*. **232**(7), 1767-1773 (2017).

